# Preoperative tumor cerebral blood flow measured by arterial spin labeling predicts enhancing tumor volume progression in gliomas: a single-center retrospective study

**DOI:** 10.1186/s12880-026-02612-9

**Published:** 2026-07-29

**Authors:** Thomas Lindner, Jens Fiehler

**Affiliations:** Department of Diagnostic and Interventional Neuroradiology, University Medical Center Hamburg-Eppendorf, Martinistr. 52, 20251 Hamburg, Germany

**Keywords:** Arterial spin labeling, Cerebral blood flow, Glioma, Glioblastoma, Perfusion imaging, Tumor progression, Enhancing tumor volume

## Abstract

**Background:**

Arterial spin labeling (ASL) enables non-invasive quantification of cerebral blood flow (CBF). The preoperative prognostic value of tumor-specific CBF for subsequent enhancing tumor (ET) volume progression in gliomas remains poorly characterized.

**Methods:**

In this study 60 glioma patients (predominantly glioblastoma, WHO grade 4) with preoperative 3T ASL MRI and paired follow-up structural MRI were analyzed. Tumor CBF, surrounding CBF, and whole-brain CBF were quantified from the segmented regions of interest. ET and surrounding non-enhancing FLAIR-hyperintense (SNFH) volume changes were calculated and correlated with preoperative perfusion data. Spearman correlations, Kruskal-Wallis tests, multivariable linear and logistic regression, and ROC analyses were performed.

**Results:**

Higher preoperative tumor CBF strongly predicted net ET volume increase (Spearman *r* = 0.531, *p* < 0.0001) and ET progression category (Kruskal-Wallis *p* = 0.0005). Mean tumor CBF was 147.7 ml/100 g/min in ET-increased versus 124.4 ml/100 g/min in unchanged and 115.8 ml/100 g/min in decreased cases. Tumor CBF remained an independent predictor after adjusting for age, scan interval, and MGMT status (linear regression *p* = 0.002; logistic regression OR = 2.02 per ml/100 g/min, *p* = 0.001). Associations with SNFH volume changes were weak and non-significant. Surrounding CBF closely matched whole-brain CBF (Pearson *r* ≈ 1.00). Tumor CBF alone yielded 5-fold CV AUC = 0.805 for predicting ET progression; the combined model (tumor CBF + ET volume) improved to AUC = 0.858.

**Conclusion:**

Preoperative ASL-derived tumor CBF appears to be an independent non-contrast imaging biomarker for early enhancing tumor progression risk in gliomas.

## Introduction

Gliomas are the most common primary malignant brain tumors in adults, accounting for approximately 80% of malignant intracranial neoplasms [1]. Glioblastoma (GBM, WHO grade 4), the most aggressive subtype, carries a dismal prognosis with median overall survival of 15–18 months despite maximal safe resection followed by temozolomide-based chemoradiation and maintenance therapy [2]. A major challenge in glioma management is the high rate of local recurrence, which occurs in > 90% of cases within 2–3 cm of the original resection cavity [3]. Early identification of patients at high risk of rapid tumor regrowth could enable personalized surveillance, intensified adjuvant therapy, or enrollment in clinical trials.

Conventional magnetic resonance imaging (MRI) remains the cornerstone of glioma assessment, relying on contrast-enhanced T1-weighted (T1CE) and T2/FLAIR sequences to delineate enhancing tumor (ET) and surrounding non-enhancing FLAIR-hyperintense (SNFH) components. While these sequences accurately depict tumor burden at a single time point, they have limited ability to predict subsequent volume dynamics or biological aggressiveness prior to treatment [4]. Baseline tumor volume alone shows only modest correlation with progression-free survival, and volumetric changes are typically assessed retrospectively after clinical or radiological progression has already occurred [5].

Advanced physiologic imaging techniques, particularly perfusion MRI, have emerged to probe tumor vascularity and neoangiogenesis which can be considered as hallmarks of glioma aggressiveness [6]. Dynamic susceptibility contrast (DSC) is a commonly used and widely studied method which requires gadolinium-based contrast agents, which carry risks of nephrogenic systemic fibrosis, gadolinium retention, and allergic reactions [7]. Arterial spin labeling (ASL) offers a non-invasive alternative by magnetically labeling arterial blood water as an endogenous tracer, enabling absolute cerebral blood flow (CBF) quantification without exogenous contrast agents [8]. ASL is particularly advantageous for longitudinal monitoring, patients with renal impairment and pediatric populations.

Multiple studies have demonstrated the utility of ASL in glioma grading. Meta-analyses report moderate-to-high diagnostic accuracy (AUC 0.85–0.92) for distinguishing high-grade from low-grade gliomas using maximum or mean tumor CBF [9, 10]. In the post-treatment setting, ASL performs comparably to DSC for differentiating true progression from pseudoprogression or treatment-related changes (AUC 0.73–0.89) [11–13]. Qualitative ASL hyperperfusion patterns have also been linked to shorter progression-free survival in newly diagnosed GBM [14]. However, most ASL research has focused on cross-sectional grading or post-therapeutic response assessment. Few studies have investigated preoperative quantitative ASL CBF as a predictor of subsequent longitudinal tumor volume changes, particularly the growth of the enhancing tumor component after initial surgery and adjuvant therapy [15–16].

The present study addresses this gap by evaluating whether preoperative tumor-specific CBF predicts enhancing tumor (ET) and SNFH volume progression in a retrospective cohort of 60 glioma patients from the UCSF Glioma dataset with matched follow-up data from the UCSF-ALPTDG cohort. The hypothesis for this study is that elevated preoperative tumor CBF reflects underlying neoangiogenic activity and would independently predict greater net ET volume increase, outperforming or complementing conventional baseline volume metrics (ET and SNFH) and clinical factors (age, MGMT promoter methylation status, and scan interval). Secondary objectives include comparing tumor CBF with surrounding and whole-brain CBF, assessing the added value of combined models and evaluating the superiority of tumor-specific over global perfusion measures.

By establishing preoperative ASL tumor CBF as a non-invasive imaging biomarker for early progression risk, this work aims to improve risk stratification, guide surveillance intensity and support the integration of ASL into routine preoperative glioma MRI protocols. By focusing on early postoperative volume changes (median interval to follow-up scan: 56 days), this study aims to identify imaging biomarkers of rapid regrowth that occur shortly after surgery and during the initial phase of adjuvant therapy.

## Materials and methods

### Study design and patient cohort

This retrospective single-center study was conducted using publicly available data from the University of California San Francisco Preoperative Diffuse Glioma MRI (UCSF-PDGM) dataset [17, 18] and the University of California San Francisco Adult Longitudinal Post-Treatment Diffuse Glioma (UCSF-ALPTDG) MRI dataset [19, 20]. The UCSF-PDGM dataset provides preoperative multimodal brain MRI from 501 patients with histopathologically confirmed WHO grade 2–4 diffuse gliomas imaged between 2015 and 2021 at a single institution. It includes standardized 3T MRI sequences with advanced diffusion and perfusion techniques, multicompartment tumor segmentations, molecular genetic data (IDH mutation, MGMT promoter methylation, 1p/19q codeletion), extent of resection, treatment history, and survival information [17].

The UCSF-ALPTDG dataset complements this by providing longitudinal post-treatment multimodal MRI from 298 patients with diffuse gliomas, consisting of two consecutive follow-up timepoints (596 scans total). Expert voxelwise segmentations delineate enhancing tumor (ET), surrounding non-enhancing FLAIR hyperintensity (SNFH), necrotic core (NCR), and resection cavities (RC) at each timepoint, with longitudinal changes in ET and SNFH annotated on subtraction sequences. Accompanying clinical data include demographics, treatment details, and genetic markers [19].

For the present analysis, 60 patients were identified with overlapping identifiers between the UCSF-PDGM and UCSF-ALPTDG datasets after matching and quality checks. The remaining patients from the UCSF-ALPTDG dataset (*n* = 238) were excluded because they lacked a corresponding preoperative ASL scan in the UCSF-PDGM dataset, had insufficient follow-up imaging quality, or did not have overlapping identifiers between the two datasets. No survival bias was introduced, as inclusion was based solely on data availability for the paired preoperative–postoperative analysis.

### MRI acquisition

All preoperative imaging (UCSF-PDGM) was performed on 3.0 Tesla scanners (GE Discovery MR750) using an 8-channel head coil. The standardized protocol included predominantly 3D sequences: T2-weighted, T2/FLAIR-weighted, susceptibility-weighted imaging, diffusion-weighted imaging, pre- and post-contrast T1-weighted images, 3D pseudo-continuous arterial spin labeling (PCASL) perfusion, and diffusion imaging [17]. The 3D pseudo-continuous ASL (PCASL) sequence used a labeling duration of 1800 ms, post-labeling delay of 2025 ms, 3D stack-of-spirals readout, TR/TE = 4675/3.0 ms, 8 averages, and 1.875 × 1.875 × 4 mm³ resolution, consistent with standard clinical 3T PCASL implementations [8, 21]. Follow-up scans (UCSF-ALPTDG) used compatible structural sequences (T1 pre- and post-contrast, T2/FLAIR) [19].

### CBF quantification and processing

Absolute CBF maps were generated using the standard single-compartment model as recommended by the ISMRM perfusion study group [8]. Processing was performed using BASIL (FSL) with standard parameters. Labeling efficiency was assumed as 0.85, blood T1 = 1.65 s at 3T, and a blood-brain partition coefficient λ = 0.9 ml/g [8, 22]. Tumor CBF was measured within delineated enhancing tumor regions of interest (ROIs) on co-registered T1 post-contrast images without areas of central necrosis. The provided ASL data was resampled to match the ROI definition of 1 mm isotropic resolution. Surrounding CBF was defined as brain perfusion excluding enhancing tumor and central necrosis (typically normal-appearing white/gray matter in peritumoral non-enhancing regions). Whole-brain CBF was defined as healthy tissue CBF including tumor CBF [23, 24].

### Volume segmentation and change assessment

ET and SNFH volumes at the first (preoperative/baseline) and second (follow-up) timepoints were obtained from expert voxelwise segmentations in the matched UCSF-ALPTDG dataset [19]. Net volume change was defined as increased volume minus decreased volume (net = Inc − Dec), based on expert voxel-wise annotations on subtraction sequences from the UCSF-ALPTDG dataset. Volume changes were categorized as Increased (net > 0), Unchanged (net = 0, i.e., no measurable increase or decrease), or Decreased (net < 0). No additional tolerance threshold was applied beyond the expert annotations.

### Clinical and molecular variables

Patient age, sex, WHO 2021 diagnosis, MGMT promoter methylation status (positive/methylated vs. unmethylated), IDH mutation status, extent of resection (GTR/STR/BX), number of surgeries, chemotherapy type (e.g., TMZ, Optune, trials), and scan intervals (days from 1st surgery/DX to 1st scan; 1st to 2nd scan) were extracted from the combined datasets [17, 19].

### Statistical analysis

Continuous variables are reported as mean ± SD or median (IQR). Spearman rank correlations assessed associations between preoperative CBF metrics and volume changes. Kruskal-Wallis tests compared tumor CBF across ET change categories, with post-hoc pairwise comparisons if significant.

Multivariable linear regression modeled ET change (dependent variable) with preoperative tumor CBF, patient age, scan interval, and MGMT status as predictors. Multivariable logistic regression predicted binary ET progression (Increased vs. not). Model performance included adjusted R² (linear) and pseudo-R² (logistic). ROC analysis with 5-fold cross-validation evaluated predictive performance of tumor CBF alone versus combined models (tumor CBF + ET volume), reporting AUC.

Analyses were performed in Python (pandas, scipy, statsmodels, scikit-learn). *P* < 0.05 was considered significant (two-sided). No multiple-testing correction was applied due to the exploratory nature.

### Ethical considerations

This study used de-identified, publicly available data from the UCSF-PDGM [17] and UCSF-ALPTDG [19] datasets hosted on The Cancer Imaging Archive and UCSF Center for Intelligent Imaging repositories. No additional institutional review board approval was required.

## Results

### Patient characteristics

The final cohort consisted of 60 patients (mean age 57.8 ± 12.4 years; 32 male, 28 female) with diffuse gliomas, predominantly glioblastoma, IDH-wildtype (WHO grade 4, *n* = 52; other diagnoses include grade 4 IDH-mutant astrocytoma, oligodendroglioma, gliosarcoma and glioma NOS). MGMT promoter methylation status was positive (methylated) in 32 cases (53.3%) and unmethylated in 20 cases (33.3%). The status was unavailable in 8 cases. Extent of resection at first surgery was gross total resection (GTR) in 18 patients (30.0%), subtotal resection (STR) in 25 (41.7%), and biopsy only in 5 (8.3%). Data were missing or not applicable in the remainder. Most patients received temozolomide (TMZ)-based therapy, with variable adjunctive treatments (Optune, nivolumab/placebo trials, dendritic cell vaccine, RRx-001). Median interval between preoperative (first) and follow-up (second) scan was 56 days (IQR 49–63).

Preoperative tumor CBF (mean 133.9 ± 24.7 ml/100 g/min) was significantly higher than surrounding CBF (111.8 ± 25.1 ml/100 g/min) and whole-brain CBF (74.2 ± 19.6 ml/100 g/min).

### Association of preoperative tumor CBF with enhancing tumor volume progression

**Fig. 1:**
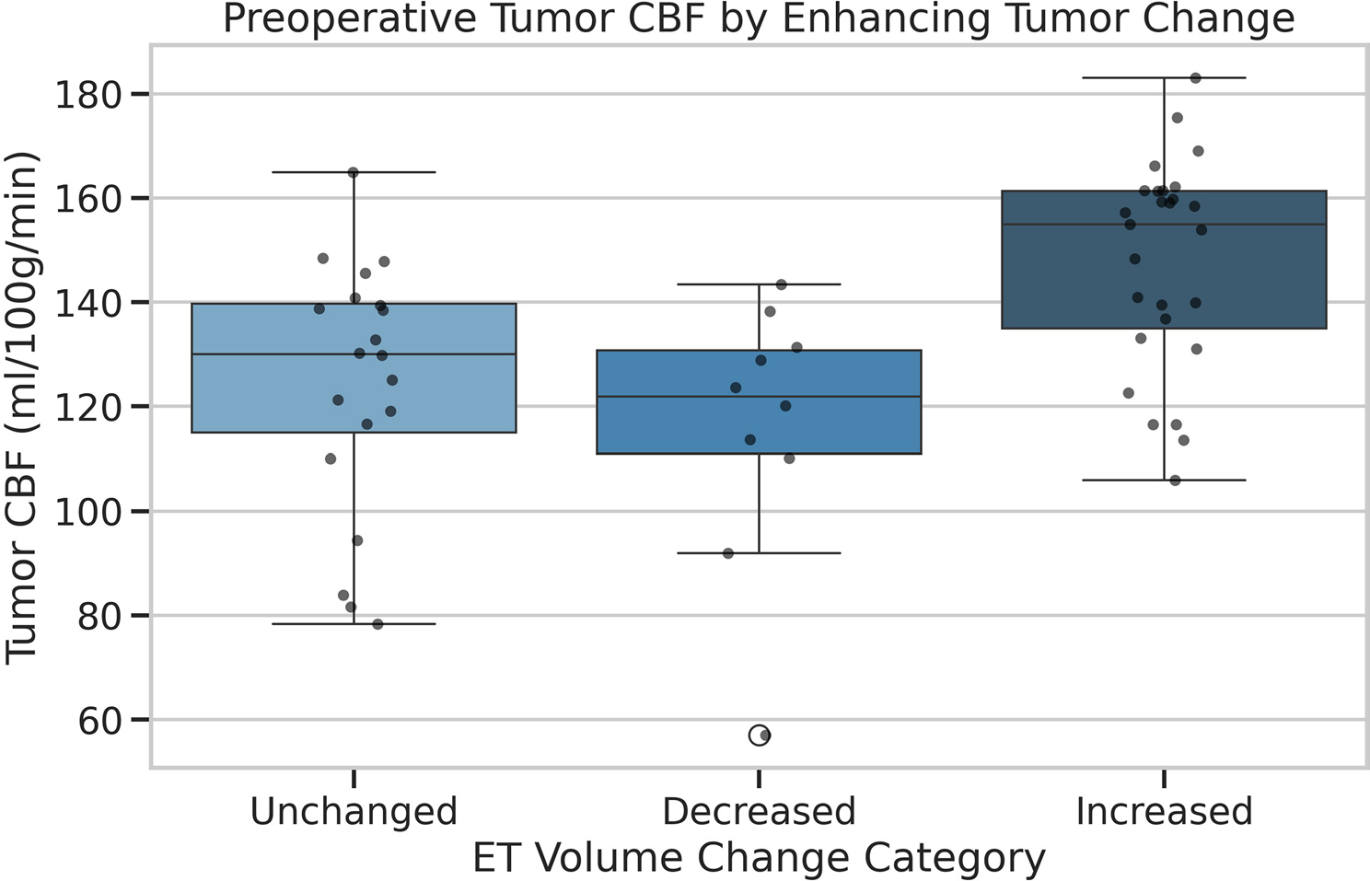
Boxplot showing preoperative tumor cerebral blood flow (CBF) stratified by enhancing tumor (ET) volume change category (Increased, *n* = 28; Unchanged, *n* = 21; Decreased, *n* = 11)

**Fig. 2:**
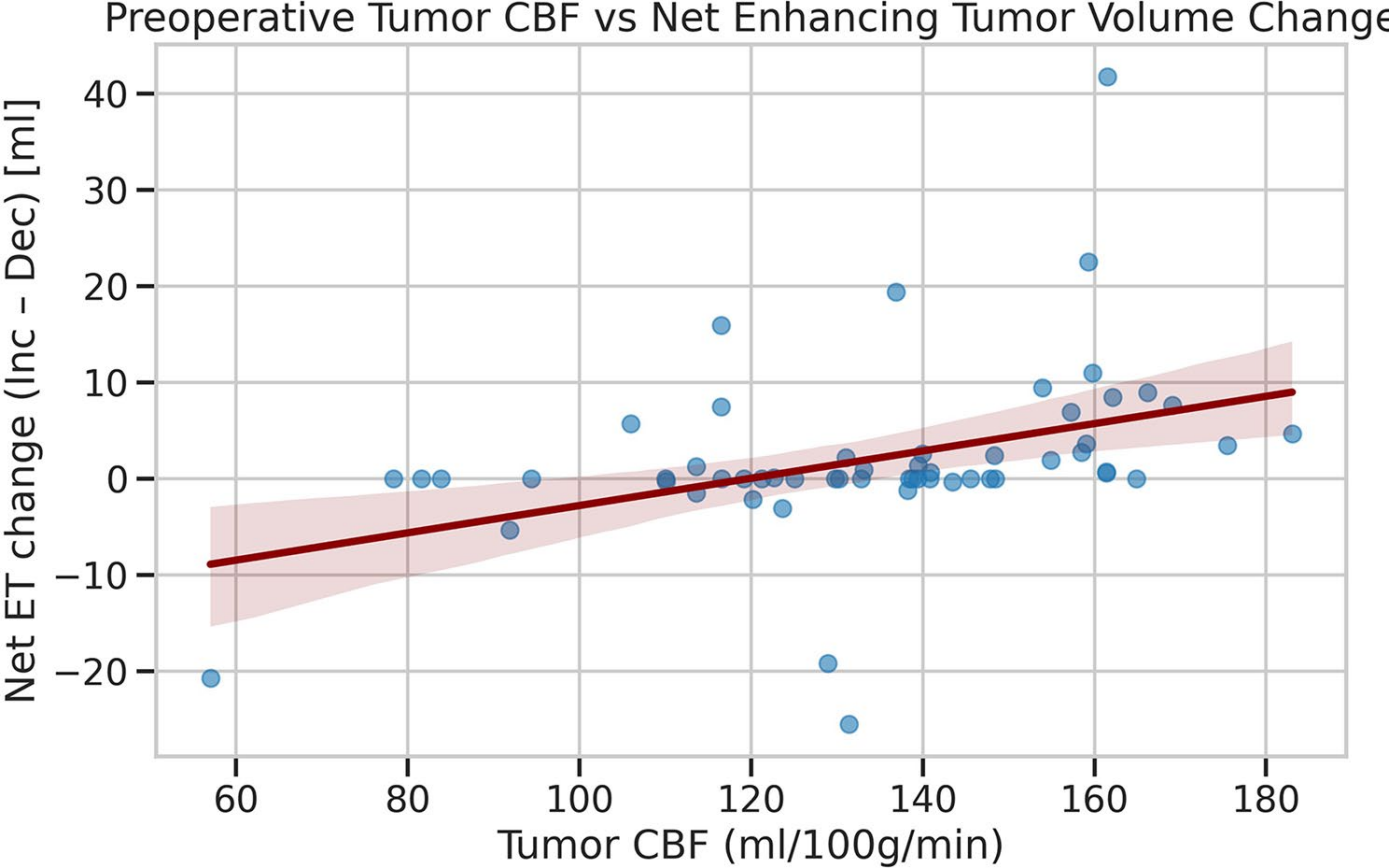
Scatterplot of preoperative tumor CBF versus net enhancing tumor volume change (increased volume minus decreased volume, in ml). Red line represents the linear regression fit across all patients (Spearman *r* = 0.531, *p* < 0.0001)

Higher preoperative tumor CBF was strongly associated with greater net enhancing tumor (ET) volume increase (Spearman *r* = 0.531, *p* < 0.0001; Fig. 2). When examined separately, tumor CBF showed a positive correlation with the volume of new enhancement (*r* = 0.452, *p* = 0.0004) and a weaker inverse correlation with the volume of resolving enhancement (*r* = − 0.261, *p* = 0.050). These secondary correlations are presented for completeness but were not the primary focus of the analysis. When stratified by categorical ET volume change, preoperative tumor CBF differed significantly across groups (Fig. 1; Kruskal-Wallis test, stat = 15.24, *p* = 0.0005). Of the 60 patients, enhancing tumor (ET) volume change was categorized as increased in 28 patients, unchanged in 21 patients, and decreased in 11 patients (Fig. 1). Mean preoperative tumor CBF was 147.7 ± 20.2 ml/100 g/min in the Increased group, 124.4 ml/100 g/min ± 24.1 in the Unchanged group, and 115.8 ± 25.4 ml/100 g/min in the Decreased group (Fig. 1).

A positive linear relationship between preoperative tumor CBF and net ET volume change is illustrated in Fig. 2.

### Multivariable analysis

Multivariable linear regression (dependent variable: net ET volume change; *n* = 52 complete cases) confirmed that preoperative tumor CBF remained a significant independent predictor after adjustment for patient age, scan interval, and MGMT methylation status (β = +1.528 ml/100 g/min increase in tumor CBF, 95% CI 0.596 to 2.459, *p* = 0.002; Table 1). The overall model explained 20.7% of variance (adjusted R² = 0.140, *p* = 0.025).

**Table 1:** Multivariable regression results predicting enhancing tumor progression

Predictor	Linear Regression (net ET, ml) β (95% CI)	*p*-value	Logistic Regression (ET Increased) OR (95% CI)	*p*-value
Tumor CBF (per ml/100 g/min)	+ 1.528 (0.596–2.459)	0.002	2.02 (1.32–3.09)	0.001
Patient Age (per year)	−0.152 (− 0.366 to 0.63)	0.162	1.00 (0.93–1.07)	0.922
Scan Interval (days)	−0.06 (− 0.221 to 0.100)	0.454	0.91 (0.84–0.99)	0.023
MGMT methylated (yes)	+ 0.725 (− 4.900 to 6.351)	0.796	0.15 (0.02–1.14)	0.067

Multivariable logistic regression predicting binary ET progression (Increased vs. not) similarly showed tumor CBF as the strongest independent predictor (OR = 2.02 ml/100 g/min, 95% CI 1.32–3.09, *p* = 0.001; Table 1). The scan interval was inversely associated with progression risk (*p* = 0.023), while age and MGMT status were non-significant in this model.

### Predictive performance

**Fig. 3:**
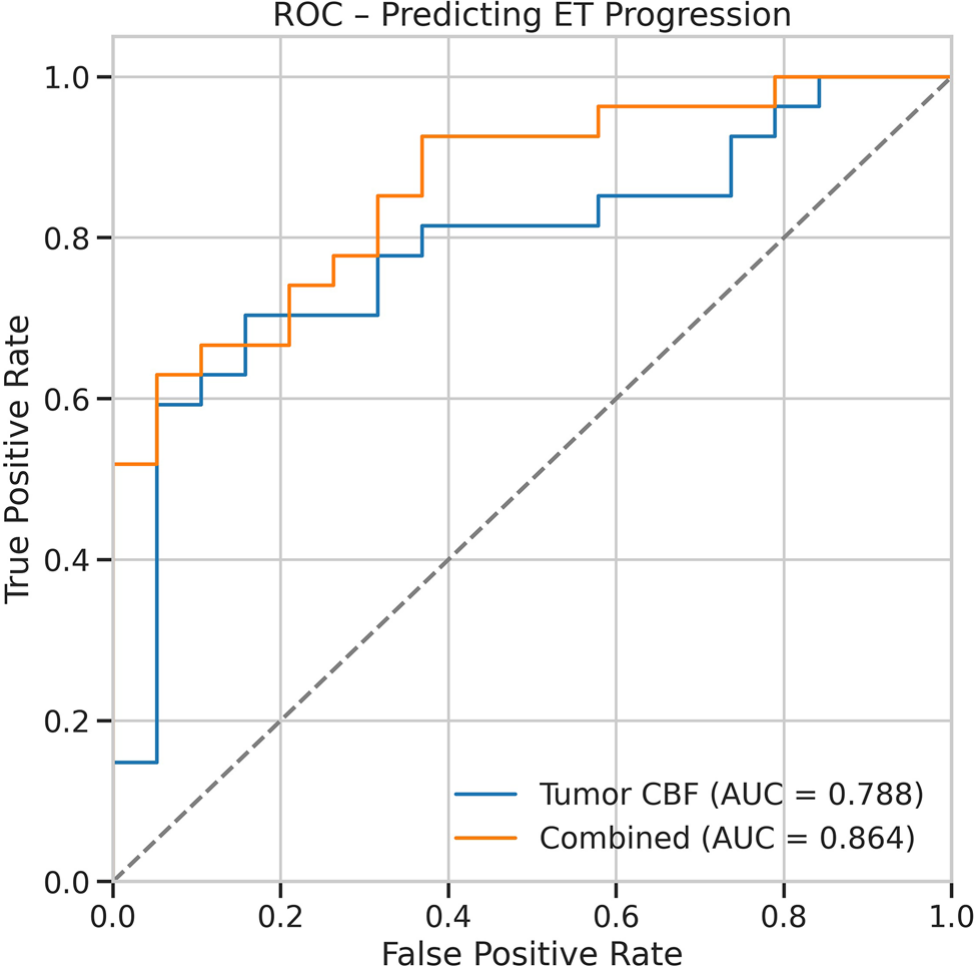
Receiver operating characteristic (ROC) curves for predicting binary ET progression (Increased vs. not). Blue: preoperative tumor CBF alone (AUC = 0.805); orange: combined model of preoperative tumor CBF + baseline (preoperative) ET volume (AUC = 0.858). Dashed line represents chance level

Receiver operating characteristic (ROC) analysis demonstrated good discriminatory ability of preoperative tumor CBF for ET progression (5-fold cross-validated AUC = 0.805). Combining preoperative tumor CBF with baseline enhancing tumor (ET) volume further improved performance (5-fold cross-validated AUC = 0.858) (Fig. 3).

No significant associations were found between surrounding CBF or whole-brain CBF and ET or SNFH volume changes after adjustment (all *p* > 0.10 in multivariable models).

## Discussion

This study demonstrates that preoperative tumor cerebral blood flow (CBF), quantified non-invasively via ASL can be used as independent predictor of subsequent ET volume progression in patients with diffuse gliomas, predominantly glioblastoma. Higher preoperative tumor CBF was robustly associated with greater net ET volume increase (Spearman *r* = 0.531, *p* < 0.0001) and with categorical progression status (Kruskal-Wallis *p* = 0.0005), with mean tumor CBF values of 147.7 ± 20.2 ml/100 g/min in progressing cases versus 115.8 ± 25.4 ml/100 g/min in regressing cases. Importantly, this association persisted after multivariable adjustment for age, scan interval, and MGMT promoter methylation status (linear *p* = 0.002; logistic OR = 2.02 per ml/100 g/min, *p* = 0.001), indicating an independent prognostic marker. In contrast, associations with surrounding non-enhancing FLAIR-hyperintense (SNFH) volume changes were weak and non-significant and surrounding CBF closely mirrored whole-brain CBF (*r* ≈ 1.00), confirming that global perfusion measures fail to capture tumor-specific vascular changes.

The observed link between elevated preoperative tumor CBF and ET progression aligns with established glioma biology. Neoangiogenesis, driven by VEGF overexpression and microvascular proliferation, is a hallmark of high-grade gliomas and correlates with aggressive behavior and rapid regrowth after resection [25, 26]. ASL-derived CBF directly reflects capillary-level perfusion and has been shown to correlate with microvascular density on histopathology [27, 28]. Our findings extend previous cross-sectional ASL studies which were primarily focused on glioma grading (meta-analyses reporting AUC 0.85–0.92 for distinguishing high- vs. low-grade tumors) [9, 10] by providing longitudinal evidence that preoperative hyperperfusion predicts early post-treatment enhancing tumor dynamics. This preoperative predictive capacity is clinically relevant, as it occurs before adjuvant chemoradiation, when treatment decisions (e.g., extent of resection, trial eligibility, intensified surveillance) can still be influenced. Our quantitative findings also extend the qualitative observations of Qiao et al. [14], who linked ASL hyperperfusion to shorter progression-free survival in newly diagnosed glioblastoma. More recently, Zeng et al. [15] and Rau et al. [16] investigated preoperative ASL CBF in high-grade gliomas and similarly reported associations between elevated perfusion and aggressive tumor behavior, though neither study examined longitudinal volume change as an endpoint. While direct comparisons are limited by methodological differences, our results are consistent with this emerging evidence linking preoperative tumor hyperperfusion to biological aggressiveness and support the extension of these findings to early volumetric progression endpoints.

Compared with conventional structural imaging, preoperative tumor CBF showed superior or complementary performance. Baseline (preoperative) ET volume was a strong univariate predictor of subsequent ET progression, consistent with prior volumetric studies [29, 30]. However, preoperative tumor CBF provided complementary value. In ROC analysis, tumor CBF alone achieved good discriminatory ability (5-fold cross-validated AUC = 0.805), while combining it with baseline ET volume further improved performance (AUC = 0.858). Baseline SNFH (FLAIR) volume, by contrast, was substantially weaker and lost significance after adjustment, suggesting that perfusion captures biological aggressiveness not fully reflected by edema or infiltrative non-enhancing signal. This superiority over FLAIR metrics for predicting enhancing-component progression is a key strength of the present work and supports the growing recognition of ASL as a non-contrast alternative to gadolinium-based DSC perfusion, especially in preoperative and longitudinal settings [31, 32].

Several limitations should be acknowledged. First, the cohort size (*n* = 60, complete-cases for multivariable regression: *n* = 52) is modest and derived from a single academic center, limiting generalizability and power for subgroup analyses (e.g., IDH-mutant vs. wildtype, MGMT-stratified). Second, the retrospective design and variable adjuvant treatments (TMZ ± Optune, immunotherapy trials, etc.) introduce heterogeneity and potential confounding, although multivariable adjustment mitigated some of these effects. Third, follow-up intervals were relatively short (median 56 days), capturing early postoperative changes but not necessarily long-term recurrence patterns or survival endpoints. Survival analysis was not powered due to censored data and treatment variability. Fourth, ASL quantification relies on fixed parameters (e.g., blood T1 = 1.65 s). Tumor tissue T1 may differ from normal brain, potentially leading to minor CBF over- or underestimation in enhancing regions. However, the standardized PCASL protocol and consistent application across patients minimize this bias, and our findings remained robust.

Despite these limitations, the magnitude of the observed effect (*r* = 0.531, independent *p* ≈ 0.001–0.002) is notable for an imaging biomarker in a heterogeneous glioma cohort and compares favorably to many DSC or radiomics studies [33, 34]. The near-identity of surrounding and whole-brain CBF further underscores the necessity of tumor-specific ROI analysis as global perfusion metrics would have completely obscured the prognostic signal.

In conclusion, preoperative ASL-derived tumor CBF appears to be a promising, non-invasive imaging biomarker for early enhancing tumor progression risk in diffuse gliomas. By identifying patients likely to exhibit rapid ET regrowth after initial surgery, this metric could inform personalized surveillance strategies, adjuvant therapy intensification or stratification in clinical trials. Prospective multi-center validation, integration with molecular classifiers (IDH, MGMT, TERT) and combination with emerging radiomics or AI-based perfusion analysis are warranted to translate these findings into routine clinical practice. 

## Data Availability

The data used in this study is available at the UCSF Dataset repository: https://imagingdatasets.ucsf.edu/dataset/2 and The Cancer Imaging Archive: [https://www.cancerimagingarchive.net/collection/ucsf-pdgm/].
